# Assessing Microstructural, Biomechanical, and Biocompatible Properties of TiNb Alloys for Potential Use as Load-Bearing Implants

**DOI:** 10.3390/jfb15090253

**Published:** 2024-08-31

**Authors:** Eyyup Murat Karakurt, Yan Huang, Yuksel Cetin, Alper Incesu, Huseyin Demirtas, Mehmet Kaya, Yasemin Yildizhan, Merve Tosun, Gulsah Akbas

**Affiliations:** 1 Brunel Centre for Advanced Solidification Technology, Institute of Materials and Manufacturing, Brunel University London, Uxbridge, London UB8 3PH, UK; eyyupmuratkarakurt@osmaniye.edu.tr; 2 The Scientific and Technological Research Council of Turkey, Life Sciences Medical Biotechnology Unit, Marmara Research Centre, Kocaeli 41470, Turkey; yasemin.yildizhan@tubitak.gov.tr (Y.Y.); mervetosun0@outlook.com (M.T.); gulsah.akbas34@gmail.com (G.A.); 3 TOBB Technical Sciences Vocational School, Karabuk University, Karabuk 78050, Turkey; alperincesu@karabuk.edu.tr (A.I.); hdemirtas@karabuk.edu.tr (H.D.); 4 Machinery and Metal Technologies Department, Corlu Vocational School, Tekirdag Namik Kemal University, Tekirdag 59830, Turkey; mehmetkaya@nku.edu.tr

**Keywords:** powder metallurgy, spacer material, load-bearing implant, corrosion performance, cytocompatibility

## Abstract

Titanium-Niobium (TiNb) alloys are commonly employed in a number of implantable devices, yet concerns exist regarding their use in implantology owing to the biomechanical mismatch between the implant and the host tissue. Therefore, to balance the mechanical performance of the load-bearing implant with bone, TiNb alloys with differing porosities were fabricated by powder metallurgy combined with spacer material. Microstructures and phase constituents were characterized with energy dispersive spectroscopy (EDS), scanning electron microscopy (SEM), and X-ray diffraction (XRD). The mechanical properties were tested by uniaxial compression, and the corrosion performance was determined via a potentiodynamic polarization experiment. To evaluate a highly matched potential implant with the host, biocompatibilities such as cell viability and proliferation rate, fibronectin adsorption, plasmid-DNA interaction, and an SEM micrograph showing the cell morphology were examined in detail. The results showed that the alloys displayed open and closed pores with a uniform pore size and distribution, which allowed for cell adherence and other cellular activities. The alloys with low porosity displayed compressive strength between 618 MPa and 1295 MPa, while the alloys with high porosity showed significantly lower strength, ranging from 48 MPa to 331 MPa. The biological evaluation of the alloys demonstrated good cell attachment and proliferation rates.

## 1. Introduction

Titanium-based alloys having porous structures are broadly used in implantology due to their distinctive properties like good corrosion resistances, including acceptable mechanical performances, and excellent cytocompatibility [1,2]. However, one of the core issues in the biomedical community is the biomechanical incompatibility between the implant and the host tissue, which may result in stress-shielding effect at implantation site. At this point, the porosity characteristics of a load-bearing implant like pore size and pore distribution should be carefully adjusted to be compatible with the biomechanical performances of the host tissue, which is beneficial in reducing stress shielding condition [3,4]. In certain biomaterial applications, pore model with optimal particle packing is recommended for improved mechanical performances [5]. Thus, porous structure within the implant material refers to a deliberate design feature to achieve desirable mechanical properties [6].

The most significant aspects of implantology are the porosity structures like pore size, distribution, and morphology. This is essential for bone ingrowth, bone infiltration, and osteogenesis [7,8]. From a nutrient and oxygen transport perspective, a porous load-bearing implant has more benefits than a stiff one, as porosity within the implant can render an adequate site, although a delicate balance is required between the pore characteristics, histological, and mechanical properties [9,10,11,12].

A porous implant material can be produced by numerous techniques like 3D printing, foaming, and powder metallurgy with or without the use of a spacer material [13]. In this study, powder metallurgy combined with a spacer material was selected to produce porous titanium-based implant materials due to their relatively cost-effective and adjustable porosity characteristics [14]. In powder metallurgy, there are several parameters that affect the packaging of particles. These are mixing/blending mechanisms, pressing pressure, sintering process temperature, and time [15]. Nevertheless, they can offer a limited porosity level on the implant material. In recent years, powder metallurgy combined with spacer material has been widely used in the production of porous implant materials as it efficiently allows the adjustment of pore characteristics. Choosing a suitable spacer material is crucial to attaining the desired biomedical and mechanical properties for titanium implants [16]. In this study, ammonium bicarbonate was selected as a spacer material to generate a wide range of porosity.

Phase constituents of titanium implants also have a strong impact on the mechanical performance. Namely, they can be alloyed with highly biocompatible elements like niobium, zirconium, tin, etc., to enhance their mechanical performance [17]. Binary TiNb alloys have recently gained a great deal of interest as they possess a β phase structure, the fraction of which depends on the niobium content and thermal treatment carried out [18]. The presence of a β phase is a vital factor for reducing elastic modulus and rendering biological passivity [19,20,21]. It has also shown a considerable effect on enhancing bone healing and bone remodeling [22,23,24]. Although clinical studies have suggested that β-phase-based Ti alloys may suffer from insufficient mechanical strength, the stiffness of β-based alloys is remarkably greater than the stiffness of bone [25,26]. The main driving force behind this research is to develop a series of TiNb alloys with balanced biomedical and mechanical properties by taking advantage of the presence of porosity and β-phase in the material.

TiNb alloys are gaining attention as implant materials due to their beneficial properties, such as low elastic modulus, and resistance to corrosion. Additionally, TiNb alloys are highly biocompatible, meaning they are less likely to cause adverse reactions in the body, making them ideal for long-term use in implants. There are various promising approaches for developing customized titanium and its alloys, particularly for implantology. For instance, Surmeneva et al. studied synthesizing Ti-10Nb (at. %) alloy using electron beam melting (EBM). They stated that EBM could be efficiently utilized for the in situ synthesis of Ti-Nb alloys [27]. Zhao et al. focused on a Ti-16Nb (wt.%) alloy produced via metal injection molding (MIM). According to this study, the Ti-16Nb alloy exhibited acceptable mechanical performance, which makes it a suitable candidate for use as an implant [28]. Chen et al. stated that niobium concentration in the titanium matrix was essential for enhancing the alloy’s biocompatibility [29]. Zhao et al. investigated a Ti–22Nb (wt.%) alloy fabricated by MIM. They found that the Ti–22Nb alloy had a good surface topography and outstanding cytocompatibility [30]. They achieved TiNb alloys with improved mechanical performances and superior biocompatibility in these investigations. Our study presents the use of powder metallurgy combined with spacer material, a relatively cost-effective technique for the fabrication of TiNb alloys. With this method, metallographic, mechanical, and histological examinations of materials can be performed much more practically and inexpensively compared to other production methods. Additionally, this study presents new insights into how this method affects the alloy’s properties, which is essential for its use in biomedical implants. In addition, this study investigates the sintering behavior of the Ti-Nb alloy.

The aim of this study is to investigate the effects of Nb content and porosity level on the microstructural, biomechanical, and biocompatible properties of Ti-xNb (x: 10, 20, and 30; at. %) alloys for potential use as load-bearing implants.

## 2. Materials and Methods

### 2.1. Production of Ti-Based Alloys

The raw metal powders employed were pure titanium (Ti, ~99.5% purity, Thermo Fisher Scientific, Waltham, MA, USA) and pure niobium (Nb, ~99.8% purity, Thermo Fisher Scientific, Waltham, MA, USA), with an average particle size of 44 µm and 35 µm, respectively. Ammonium bicarbonate (NH_4_HCO_3_, #1066-33-7, Thermo Fisher Scientific, Waltham, MA, USA) was employed as a spacer material (SM). The chemical formulas are given in Table 1. Ti and Nb raw metal powders with the nominal compositions shown in Table 1 were blended, using a powder blending apparatus for 10 h. NH_4_HCO_3_ was added to generate extra porosity. Afterwards, powder mixtures were placed in a cylindrical steel die of Ø10 mm × 15 mm in dimension, and then briquetted under 300 MPa at a compaction speed of 0.1 mm/min, following ASTM E9-22 (Standard Test Methods of Compression Testing of Metallic Materials at Room Temperature. ASTM International: West Conshohocken, United States, 2022). Low porous briquettes were sintered at 1200 °C for 6 h, while highly porous briquettes were first heated up to 180 °C for 2 h to eliminate NH_4_HCO_3_, followed by sintering at 1200 °C for 6 h. The furnace was operated at a heating rate of 5 °C per minute within an argon gas atmosphere. Subsequently, the samples were gradually cooled down to 200 °C. After the metallurgical preparation, each sintered sample was immersed in a Keller’s solution (190 mL H_2_O, 5 mL HNO_3_, 3 mL HCl, and 2 mL HF) [31].

### 2.2. Microstructural and Biomechanical Characterization

Microstructural analysis was done by Zeiss Supra 35 microscope (Zeiss Group, Oberkochen, Germany). XRD spectra was performed by a Bruker D8 Advance Cu-Kα source, using a step size of 0.02°, an acquisition time of 1 s, and an angle range of 30° to 80°. The uniaxial compression test was done on a universal tensile-compression testing machine (Zwick/Roell Z600). The general porosity (ɛ) was determined from following Equations (1)–(3) [32].

(1) $$\rho_{o}=\frac{m_{Ti}+m_{Nb}}{V_{Ti}+V_{Nb}}$$

(2) $$\rho =\frac{m}{V}$$

(3) $$ɛ=\left(1-\frac{\rho }{\rho_{o}}\right)\times 100$$

where ρ_o_ is the theoretical density; m_Ti_, the mass of titanium; V_Ti_, the volume of titanium; m_Nb_, the mass of niobium; V_Nb_, the volume of niobium; ρ, the alloy density after sintering, m, the alloy mass; v, the alloy volume; ɛ, the general porosity.

Corrosion performance was assessed via potentiodynamic polarization experiments in Hanks’ Balanced Salt Solution (Thermo Fisher Scientific, #J67763, MA, USA). Each potential scan was performed at a scan rate of 0.01 V/s vs. open circuit potential from −0.75 to 1.25 V.

### 2.3. In Vitro Testing of TiNb Alloys

In vitro tests were performed by MTT cell viability assay, plasmid-DNA interaction assay, fibronectin adsorption, and SEM analysis showing the cell morphology to assess the individual effect of niobium content and the general porosity level in Ti based composites on their biocompatibility in comparison with the reference TiGR4 material.

Cell viability and proliferation: In vitro studies for Ti based alloys carried out using mouse fibroblast NCTC clone 929 (L929) and human bone osteosarcoma (Saos-2) cell lines purchased from American Tissue Culture Collection (ATCC) (Manassas, VA, USA). The alloy disc samples with diameter in 8 mm and thickness in 2 mm autoclaved at 120 °C for 30 min were extracted at a ratio of 0.2 g/mL in DMEM including 10% Fetal Bovine Serum (FBS) at 37 °C and 120 rpm for 72 h.

MTT Assay: The cell lines cultured in DMEM including 1% Antibiotic-Antimycotic solution (Gibco, #15240-062, Carlsbad, CA, USA) and 10% FBS (Gibco, #10500-064, New York, NY, USA) were seeded into the each well of the 96-well plate and cultured at 37 °C and 5% CO_2_ for 24 h. Following day, the medium was aspirated from the each well and the sample extracts were added in to the wells and then the plate was placed in the incubator at 37 °C and 5% CO_2_. After each incubation periods of 1-day, 3-day, and 7-day, 3-(4,5-Dimethyl-2-thiazolyl)-2,5-diphenyl-2H-tetrazolium bromide (MTT) (Sigma-Aldrich, #M5655, St. Louis, MO, USA) (0.5 mg/mL) in DMEM was added on the cell monolayers incubated for 4 h. After that, the formazan crystals occurred from MTT agent due to the metabolic activity of alive cells was dissolved with adding dimethyl sulphoxide (DMSO) into the wells and then the plate was placed in a shaker at room temperature for 2 h. The optical density (OD) obtained from samples and the controls was determined at 570–630 nm wavelength with a Microplate reader (BioTek Instruments, Inc., Winooski, VT, USA). The absorbance in the each well measured upon application of alloys’ extracts was normalized to the control absorbance value and the cell viability was calculated as the percentage of the control.

Live-Dead Assay was carried out according to the protocol in the product test kit (Molecular Probes #L3224, Eugene, OR, USA). It is based on determination of the alive cells stained with green fluorescent calcein AM (ex/em ~495 nm/~515 nm) and the membrane damaged dead cells stained with red fluorescent Ethidium homodimer, EthD-1 (ex/em ~495 nm/~635 nm). The cells were cultured in the wells of the 96 well plate and following day, the cells exposed to the sample extracts as described before. After washing the cells with DPBS, calcein AM (2 µM) and EthD-1 (4 µM) in 100 µL of DPBS was added then incubated at 25 °C for 30–45 min. The images of the cell monolayers were taken at 10× magnification using a fluorescence microscope (Leica DMI 6000, GmbH, Wetzlar, Germany).

Fibronectin Activity—Cell Adhesion: The fibronectin (FN) adsorption potential of the TiNb alloy disks and the reference TiGR4 disks were examined by a modified enzyme-linked immunosorbent assay (ELISA). The discs were placed into the wells of the ELISA plates which were coated with 1% Bovine serum albumin (BSA in PBS) and then incubated at 37 °C for 2 h. FN (2.5 µg/mL) in PBS (Gibco, #14200-67, Carlsbad, CA, USA) was added on the alloy disks in the wells and the plate was incubated for 2 h. Following that, the primer monoclonal anti-FN antibody (1:10,000) (Sigma #SAB4200760, St. Louis, MO, USA), in 550 µL was added into each well of the plate incubated at 4 °C for overnight. After that, the secondary antibody, goat anti-mouse immunoglobulin G conjugated with horse radish peroxidase (1:30,000) (Sigma #A2554, St. Louis, MO, USA) was added and incubated at 37 °C for 30 min. Then the stop solution, 3,3′,5,5′-tetramethylbenzidine (TMB, Sigma #860336, St. Louis, MO, USA) in 200 µg/mL was added in to the wells. Following the formation of blue colour was observed, 275 µL of 2 M H_2_SO_4_ was added into the wells. The experiments were repeated in triplicate. The optical density was measured spectrophotometrically at 450 nm.

Plasmid DNA Interactions: To evaluate the interactions between the alloy discs’ extracts and DNA of the plasmid, pBOS-H2B-GFP plasmid (5.8 kb, BD Pharmingen William Saunders, NJ, USA) grown in *E-coli* was purified using a Machery Nagel DNA isolation kit (Macherey-Nagel GmbH & Co. KG, #740235, Dueren, Germany). The alloy discs’ extracts with 200 ng of plasmid DNA in ddH_2_O were incubated at room temperature for 16 h. 1% agarose gel electrophorese for the samples and controls was run at 100 V for 1 h using TAE buffer. After that, ethidium bromide was used for staining the gel, and the band images were taken with ChemiDoc imaging system (BioRad, Hercules, CA, USA).

Scanning Electron Microscope: The examined L929 and Saos-2 cells were seeded on the discs of alloy and TiGR4 reference samples and the plate was incubated at 37 °C and 5% CO_2_ for 1 day and 7 days. Fixative 2.5% glutaraldehyde solution for the cell monolayers were added at 25 °C for 1 h and 60%, 70%, 80%, 90%, and 100% of ascending ethanol concentrations were used for dehydratation at 20 min for each then they were left to dry at 25 °C for 24 h. Finally, the L929 and Saos-2 cells images on the alloy discs were taken with a Field Emission Gun–scanning electron microscope (FEG-SEM), (QUANTA 250, FEI, OR, USA).

Statistical Analysis: The results of the MTT and the fibronectin adsorption assays were statistically analysed using GraphPad Prism 8.0.2 and the significant differences were determined by using one-way ANOVA and two-way ANOVA Tukey’s multiple comparisons test within 95% of confidence interval, (n = 3, *p* < 0.001).

## 3. Results

### 3.1. Material Characterization

**Figure 1:**
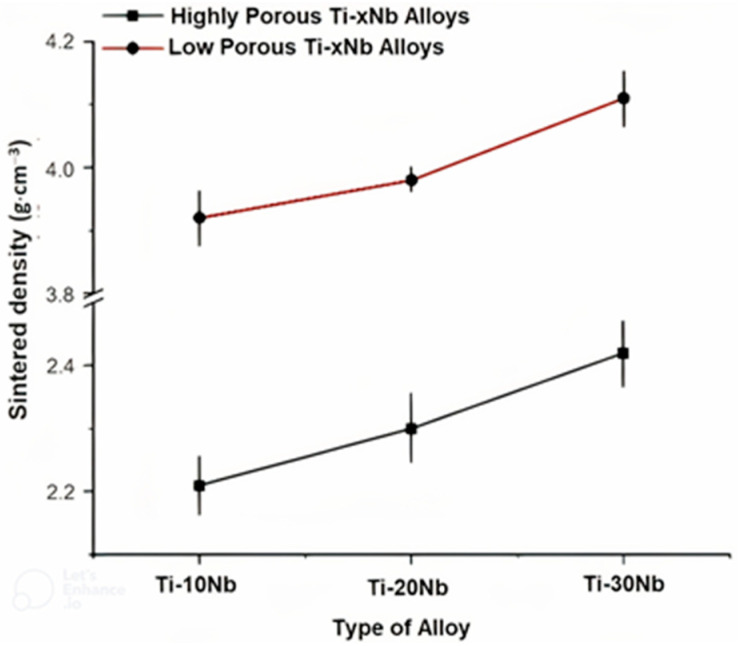
Sintered density of the Ti based alloys.

The theoretical density and bulk density are shown in Figure 1. The theoretical density for binary Ti-10Nb, Ti-20Nb and T-30Nb alloys were 4.91, 5.32 and 5.74 g·cm^−3^, respectively. The bulk density values calculated using Equation (3) for the alloys with low porosity were 3.92 for Ti-10Nb, 3.98 for Ti-20Nb, and 4.11 g·cm^−3^ for Ti-30Nb, respectively while those for the high porous alloys were 2.21 for Ti-10Nb, 2.30 for Ti-20Nb, and 2.42 g·cm^−3^ for Ti-30Nb, respectively. The sintered density increased with the increased concentration of Nb as shown in Figure 1.

**Figure 2:**
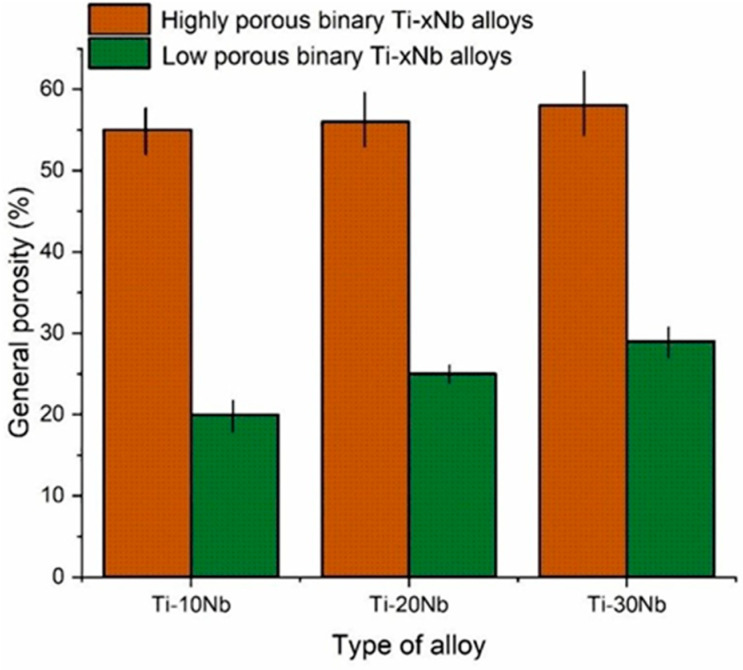
The graph showing general porosity of the Ti based alloys.

The general porosity graph is shown in Figure 2. The general porosity for the highly porous TiNb alloys was between 55% and 58% while the ranges for the low porous TiNb alloys was between 20% and 29%. It should be noted that there was no trace of NH_4_HCO_3_ residues and NH_4_HCO_3_ was replaced by pores in the microstructures of the highly porous TiNb alloys.

Hence, this result revealed NH_4_HCO_3_ used as spacer material was effective in generating porosity. Although the concentration of Nb also exhibited certain impact on the porosity as shown in Figure 2. Adding spacer material is considered a critical approach for obtaining desirable general porosity with higher efficiency compared to doping Nb [33,34].

**Figure 3:**
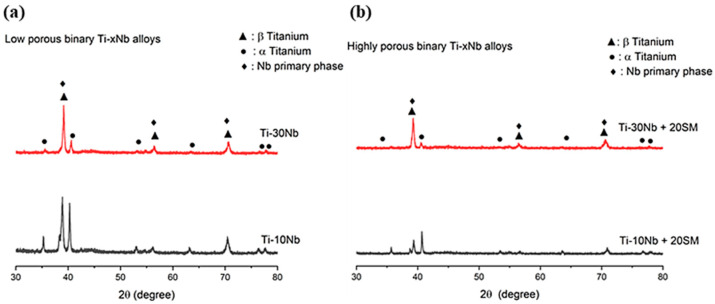
XRD spectra of the (**a**) Ti-10Nb and Ti-30Nb, (**b**) Ti-10Nb + 20SM and Ti-30Nb + 20SM alloys.

XRD spectra for the low and high porosity Ti-10Nb and Ti-30Nb alloys are given in Figure 3. hcp α, bcc β and primary Nb phases were detected from the spectra. As expected, the addition of spacer material showed no impact on the development of the phase constituents of the alloys. The volume fractions of hcp α, bcc β and primary Nb phases for low porous Ti-10Nb alloy were 93%, 3%, and 4%, while those for low porous Ti-30Nb alloys were 19%, 40%, and 41%, respectively. The formation of β phase is attributed to the addition of Nb. Nb is an effective β phase stabiliser due to its ability to increase the lattice parameter of titanium and promote the formation of bcc β in hcp α [35,36,37,38]. In addition, when examining the X-ray pattern, the peak intensities of low porous TiNb alloys were relatively greater than that of highly porous alloys, which implied that the crystallization feature of low porous TiNb alloys enhanced depending on decreasing general porosity in the alloys [39].

**Figure 4:**
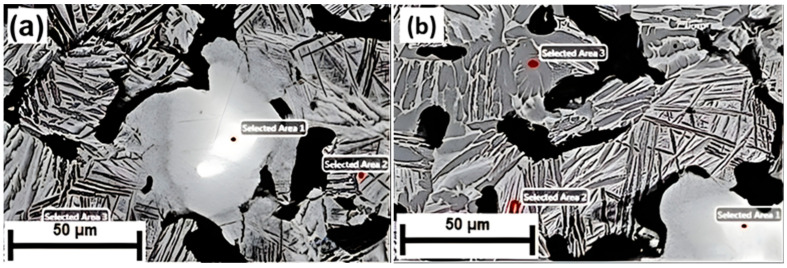
SEM micrograph indicating EDS points (**a**) Ti-30Nb, (**b**) Ti-10Nb + 20SM alloys.

EDS analysis for Ti-30Nb and Ti-10Nb + 20SM alloys is displayed in Figure 4. From Ti, and Nb concentration perspective, the remarkable alterations were observed due to phase transformations during sintering. EDS peaks detected were Ti-Kβ, Ti-Kα and Nb-L. A summary EDS analysis is given in Table 2. EDS results revealed that there was limited Nb dissolution in α phase, which is exhibited in the dark-grey areas, whereas signficant Nb dissolution was found in the β phase as shown in the light-grey areas. The bright-white area was found to be as a primary Nb phase. It can be seen from the SEM micrographs in the figure, the α and the β phases exhibited irregular shapes and boundaries, most likely being caused by the inhomogeneity of powder metallurgy.

**Figure 5:**
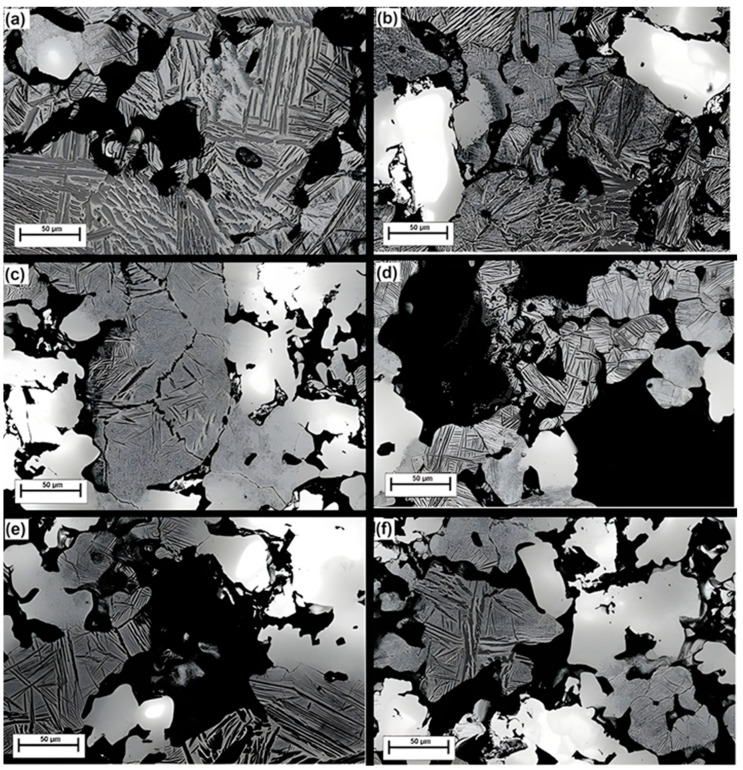
SEM micrographs of the (**a**) Ti-10Nb, (**b**) Ti-20Nb, (**c**) Ti-30Nb (**d**) Ti-10Nb + 20SM, (**e**) Ti-20Nb + 20SM, (**f**) Ti-30Nb + 20SM).

The SEM micrographs in Figure 5 show general features of the microstructures of the TiNb alloys, with hcp α, bcc β phases as primary structural elements, plus some primary Nb cores as confirmed by XRD analysis in the bright areas. The presence of Nb cores implied that the sintering temperature chosen for this study was inadequate as the sintering temperature selected was slightly low. Indeed, previous work showed that the ratio α/β was largely dependent on sintering temperature [40]. Specifically, Rao et al. found that elevated sintering temperatures affected the dissolution of Nb particles, resulting in the suppression of the Widmanstatten morphology [41].

### 3.2. Compressive Performance

**Figure 6:**
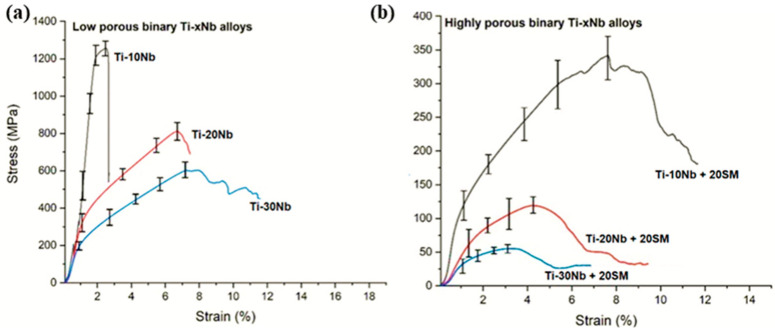
Compressive stress–strain curves showing mechanical performance as a function of (**a**) Nb concentration and (**b**) the presence of the spacer material.

The stress–strain slopes are given in Figure 6 as a function of the presence of spacer material and Nb concentration. Typical mechanical properties of the elaborated TiNb alloys are listed in Table 3. Mechanical properties varied largely among the alloys with different Nb concentration and porosity. The ultimate compressive strength with a general porosity in the range of 20–29% was 1295, 802, and 618 MPa for Ti-10Nb, Ti-20Nb and Ti-30Nb alloys, respectively, whilst those for Ti-10Nb, Ti-20Nb and Ti-30Nb with a general porosity between 50% and 59% were 331, 127, and 48 MPa, respectively. As expected, there was an inversely proportional relationship between ultimate compressive strength and general porosity. Indeed, porosity tends to cause crack initiation during compressive tests, leading to a significant decrease in the ultimate compressive strengths and yield strengths of highly porous Ti-Nb alloys [42].

Another key parameter that affected the mechanical testing was the alloy concentration related microstructure of the alloys. The increase in Nb concentrations from 10% to 30% slightly decreased the strength values due to the formation of β phase, which was confirmed by other studies [19]. Collectively, as phenomenon observed in general porosity formation, alloying Nb with the Ti had a minimal impact on the mechanical properties of the alloys. However, adding spacer material had a serious impact.

### 3.3. Corrosion Performance

**Figure 7:**
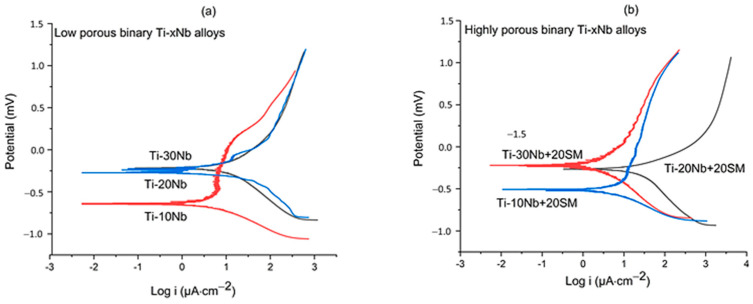
Representative polarisation curves of the (**a**) TixNb alloys and (**b**) TixNb with spacer material.

Figure 7 shows typical Tafel polarisation curves of the alloys. The corrosion potential (Ecorr) values for the low porous Ti-10Nb, Ti-20Nb, and Ti-30Nb alloys were −0.64, −0.26, and −0.21 mV, respectively; the corresponding current density (icorr) values were 0.65, 1.63, and 1.77 µA·cm^−2^, respectively. The low porous Ti-30Nb alloy with a porosity of 25% displayed the minimum corrosion rate, with an Ecorr of −0.21 mV, and icorr of 1.77 µA·cm^−2^ while the maximum corrosion rate was observed in the low porosity Ti-10Nb alloy with a porosity of 21%, having an Ecorr of −0.64 mV and icorr of 0.65 µA·cm^−2^. On the other hand, the Ecorr figures for the highly porous Ti-10Nb, Ti-20Nb, and Ti-30Nb alloys were −0.51, −0.27, and −0.22 mV, respectively and icorr values of 0.84, 1.45, and 0.49 µA·cm^−2^, respectively. It showed that the increase in Nb concentration had no negative impact on the corrosion resistance of the material. In fact, an apparent increase in the Ecorr with increasing niobium concentration observed in Figure 7a suggested that Nb addition enhanced the corrosion resistance, which is in agreement with the observation of the minimum corrosion rate in the Ti-30Nb alloy in this work and previous investigations [43,44,45,46]. Figure 7b shows that adding a spacer material did not significantly change the corrosion rate of TiNb alloys having high porosity.

### 3.4. Biocompatibility of TiNb Alloys

**Figure 8:**
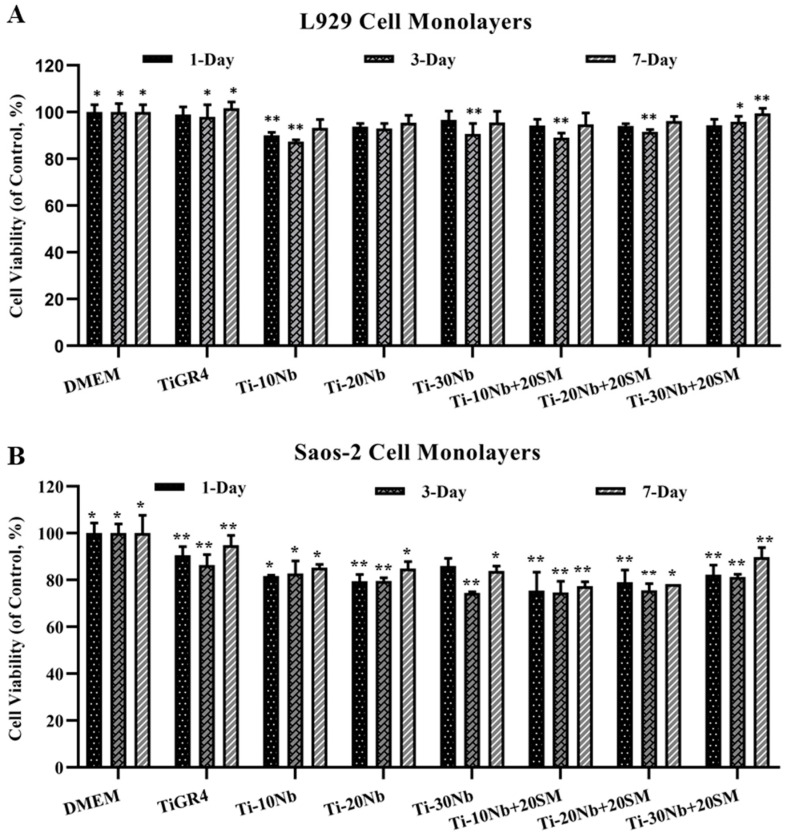
The percentage of cell viability of (**A**) L929 and (**B**) Saos-2 monolayers exposed to the Ti based alloys and the reference TiGR4 disc extracts for 1 day, 3 days, and 7 days were determined by MTT assay. Data represent mean ± SD, n = 3. *, ** for *p* < 0.05.

The biocompatibility of TiNb based alloys’ extracts and TiGR4 (reference disc) extracts were investigated using L929 and Saos-2 cell monolayers exposed for 1 day, 3 days and 7 days and their effects on the cell viability is presented in Figure 8. Different incubation times did not lead to any dramatic influence on the cell viabilities used in this study, which demonstrated that all alloy compositions did not elicit cytotoxic response on the used cell lines. In this study, the viability of the L929 and Saos-2 cell lines exposed to the extracts of TiNb alloys was found to be in a range of 87% to 96% and 73% to 90%, respectively (*p* < 0.05). The highest cell viability of L929 was 99.42% obtained from the application of highly porous binary Ti-30Nb alloy with general porosity of 58% for 7 days. This result was even higher than the cell viability ranges obtained from TiGR4 treatment. This behaviour demonstrated that cell growth and proliferation i.e., biocompatibility improved with highly porous structures within these alloys. This might be due to the presence of highly porous structures having a specific effect: it encouraged and supported the attachment and proliferation of the cells. The lowest L929 cell viability level at 87.35% was obtained from the treatment with low porous Ti-10Nb alloys with 20% of the general porosity. This result was almost at the same level of the cell viability upon exposure to the TiGR4 for all incubation times. In the case of cell viability of Saos-2 cell line affected from TiNb based samples’ extracts was similar. Here, the treatment with highly porous Ti-30Nb alloy with general porosity of 58% for 7 days resulted in the highest Saos-2 cell viability rate of 89.71% whereas exposure to the highly porous Ti-10Nb alloy with general porosity of 55% for 1 day had the lowest cell viability level of 70%. Although all TiNb alloys were found to be biocompatible, they showed significant differences in their cell viability. However, these results indicated that all pore characteristics provided an adequate site for the infiltration and proliferation. According to this concept, all alloy compositions had excellent cell viability for all the incubation times. Alloying niobium with titanium and adding a spacer material improved the cell viability. Collectively, the results revealed that the TiNb alloys investigated in this study met the standards of biocompatibility required for load-bearing implant and reached at least of 70% as specified by International Organization for Standardization (ISO) Biological evaluation of medical devices Part 5: tests for in vitro cytotoxicity. 3 ed. 2009, Geneva, Switzerland (ISO 10993-5).

**Figure 9:**
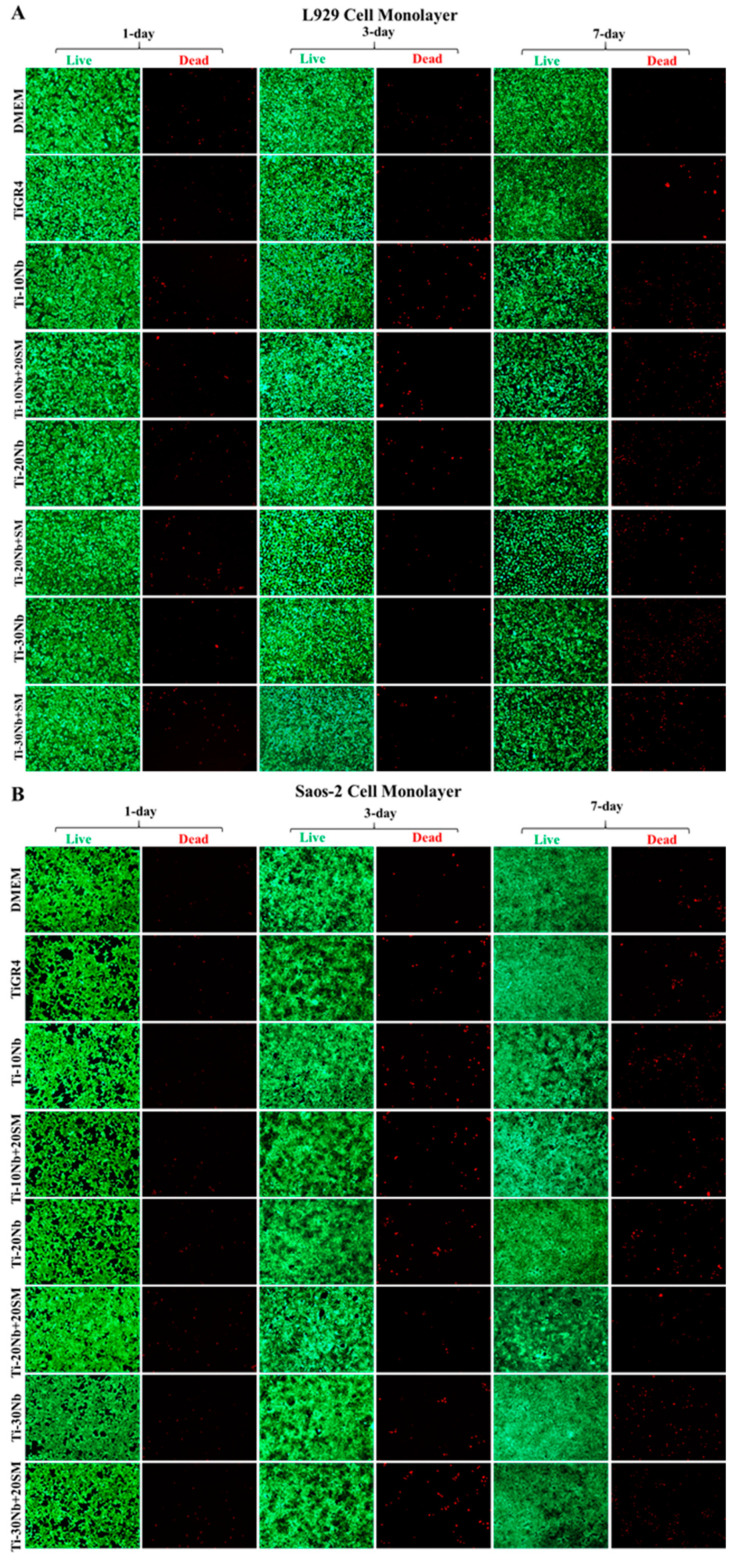
The images of (**A**) L929 and (**B**) Saos-2 cell monolayers upon exposure to the Ti-Nb based alloy extracts for 1-day, 3-day, and 7-day were taken by fluorescence microscope with 10× magnification.

L929 and Saos-2 cells were exposed to the TiNb based alloy extracts during 1 day, 3 days, and 7 days and then the live-dead cells were determined by staining with the fluorescent agents (calcein AM and EthD-1) (Figure 9). In addition to the calorimetric MTT assay, following the 24 h treatment with the TiNb alloys and the controls, alive and dead L929 and Saos-2 cells were qualitatively observed as seen in Figure 9. These fluorescence images from each treatment were also confirmed the high level of cell viability observed with the high green fluorescence intensity seen in the both cell monolayers.

**Figure 10:**
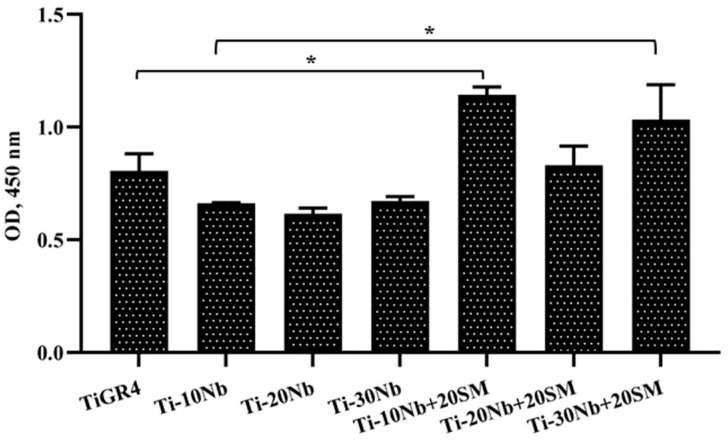
Adsorption of Fibronectin on Ti-Nb alloy disks was determined after 2 h incubation at 37 °C in a 5% CO_2_ atmosphere by ELISA method. Data represent mean ± SD, n = 3. * for *p* < 0.05.

Adsorption of fibronectin (FN) on the treated TiNb alloys is shown in Figure 10. The fibronectin adsorption capacity was not significantly affected with increased niobium content from 10% to 30% in titanium-niobium alloys. However, higher porosity dramatically increased the FN adsorption capacity of the highly porous TiNb alloys.

**Figure 11:**
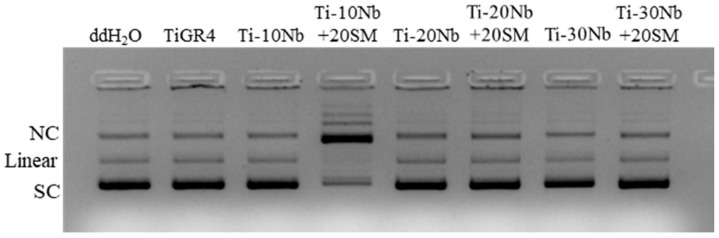
Plasmid-DNA interaction assay for TiNb based alloys. Migration pattern of plasmid DNA incubated with extracts of the TiNb based alloys and TiRG4 reference materials. The bands are labeled as NC: Nicked circular, SC: Supercoiled, ddH_2_O served as a negative control.

To comprehensively investigate biocompatibility, in vitro plasmid-DNA interaction was also assessed with genotoxicity. The pattern of plasmid DNA is shown in Figure 11. According to this, highly porous Ti-10Nb alloy exhibited Nicked Circular (NC) DNA plasmid form while the other alloys displayed Super Coiled (SC) DNA form, including TiGR4 and negative control. These achieved results showed that TiNb alloys did not cause any DNA damage.

**Figure 12:**
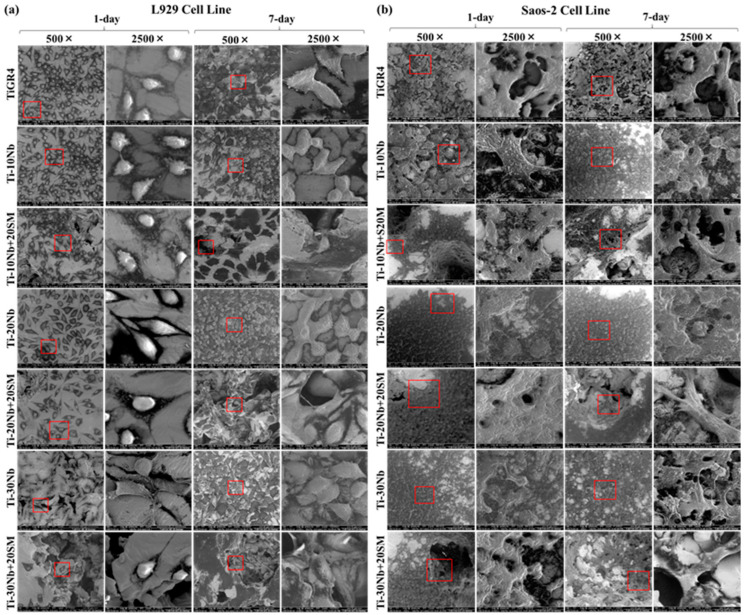
SEM images of viable (**a**) L929 cells and (**b**) Saos-2 cells on the TiGR4 and Ti-Nb based alloys for 1-day, and 7-day with 500× and 2500× magnification.

SEM micrographs showing the viability of L929 and Saos-2 cells upon exposure to the TiNb based alloys’ extracts for 1 day and 7 days are shown in Figure 12. SEM micrographs were taken at 500× magnification; Images at 2500× magnification images taken from the red square area of the 500× images. The presence of viable L929 cell line suspended on the substrate was normal because L929 cell line came detached from the surface by becoming round during the division. As foreseen, the surface roughness of the highly porous TiNb alloys with an average general porosity of 56% was higher than those of low porous binary TiNb alloys with an average general porosity of 24%. However, there were no big differences between the cell attachment and proliferation of cell viability assay. In conclusion, the cell lines were well attached to the surface of all the alloy compositions. Such condition indicated good integration with the substrate.

## 4. Discussion

Adding spacer material was crucial factor to generate a wide range of porosity. The addition of 20% weight percentage of spacer material in this work generated substantially greater pore size and volume fraction than the alloys without spacer material. In addition to enhanced porosities, the surface roughness was observed to have increased in highly porous TiNb alloys in comparison with low porous TiNb alloys. Such condition is a desirable feature for implantology because rougher surfaces can increase the bone-implant interface interaction. Natural bone generally contains a porous structure ranging from 10% to 85% according to the type of gender and age of bone. The general porosities formed in all alloys in this work fall within the range. XRD results showed that increasing Nb concentration from 10% to 30% increased the formation of the β phase despite of insufficient sintering conditions. This finding was consistent with research conducted previously [47]. Thus, the phase constituents of the alloys were able to be adjusted by adding niobium to obtain appropriate microstructures.

TiNb phase diagram shows us that there are two main phases; At this regard, β and α phases were detected by X-ray patterns of examined TiNb alloys. Moreover, According to EDS result, Table 2 showed us that Nb dissolution was high in the β phase while it was limited in the α phase. In this condition, X-ray patterns and EDS results were consistent with each other. Increased general porosity led to inhomogeneous structure as the diffusion path between particles increased, resulting in more undissolved Nb particles. This was consistent with the research conducted by Rodriguez group [48].

Mechanical testing results suggested that the mechanical performance of TiNb alloys could be adjusted by modifying the general porosity level in the alloys. The general porosities obtained in the alloys were beneficial in reducing the risk of the stress-shielding condition. The highly porous alloys with general porosities in the range of 55% to 58% displayed mechanical performance closer to the mechanical properties of human bone, compared to the low porosity alloys with general porosities between 20% and 29%.

The development of biomedical implants for clinical applications has been a critical challenge for material research, partly due to the need for defeat their adverse effects on host health. The biomechanical and biophysical characteristics of implants have direct influence on biocompatibility due to the interaction between implant material and targeted tissue. The present study was therefore designed to develop biomaterial from TiNb alloys with optimized biomechanical properties in parallel to improved biocompatibility. Previous studies on the biocompatibility Ti-based alloys containing 25% of Nb using Saos-2 cells, human osteosarcoma cell line (MG63) resulted in improved cell viability and cell adherence relative to the control, reference material. In another study, the biocompatibility of Ti-26Nb alloy using human skin fibroblast cells was investigated by assessing cell number, growth rate, the cell proliferation and cell adherence proteins (collagen III and vascular endothelial growth factor (VEGF)) which were improved compared the control reference Ti based alloy [49,50]. The findings from the previous studies supported the results of biological evaluation in this study. Overall, the Nb containing Ti alloys with homogenous porosity found to be good cytocompatibility, adherence potential, osteointegration capacity demonstrated promising candidate for orthopedic and dental applications due to their improved mechanical properties and biological performance [51].

## 5. Conclusions

An experimental study has been carried out on microstructural, biomechanical, and in vitro biocompatibility performance of a series of TiNb alloys. The alloys were fabricated by powder metallurgy in combination with the spacer technique, and two groups of TiNb alloys with a porosity in the range of 2–29% and 55–58% were successfully prepared. The microstructure, mechanical properties and in vitro biocompatibility performance were characterised to assess their potential for biomedical applications as orthopedic implants. XRD analysis showed that all alloy alloys were consisted of α and β phases as matrix with a limited amount of primary niobium phase. Experimental results showed that the addition of spacer material was effective in generating high porosity, which allows the adjustment of mechanical properties and biological performance. As expected, TiNb alloys with low porosity showed higher ultimate strength than those with high porosity. For example, Ti-10Nb alloy with low porosity showed an ultimate strength of 1295 MPa, in comparison to 331 MPa for the high porosity Ti-10Nb alloy. According to electrochemical polarization tests, TiNb alloys with different porosities possessed good corrosion performance, which is essential for implantology. According to in vitro studies, Ti and Nb were not cytotoxic or allergic at tested conditions and reduced the risk of adverse reactions. TiNb alloys achieved in this work were determined to be 87% to 96% for L929 and 73% to 90% for Saos-2 cell lines. Biological evaluations of the alloys studied met the biocompatibility criteria required for orthopedic biomaterial use. In Addition, TiNb alloys had a good corrosion performance in Hanks’ Balanced Salt Solution, which is crucial for maintaining the structural integrity of the alloys. In summary, our findings were supported by research conducted in the past. Collectively, the alloys under the present study satisfied the cytocompatibility criteria required for clinical applications of load-bearing implants owing to acceptable strength properties, good corrosion performance, and outstanding biocompatibility.

## Figures and Tables

**Table 1:** The chemical formulas of the alloys used (SM: spacer material).

Alloy Group	Chemical Formula (at. %)
Low porous alloys	Ti-10Nb
	Ti-20Nb
	Ti-30Nb
Highly porous alloys	Ti-10Nb + 20 (wt.%) SM
	Ti-20Nb + 20 (wt.%) SM
	Ti-30Nb + 20 (wt.%) SM

**Table 2:** Individual EDS points of the Ti based alloys.

Alloys	Area 1 (at. %)		Area 2 (at. %)		Area 3 (at. %)	
	Ti	Nb	Ti	Nb	Ti	Nb
Ti-30Nb	0.2	99.80	80.24	19.76	96.48	3.52
Ti-10Nb + 20SM	13.72	86.28	94.14	5.86	98.26	1.74

**Table 3:** Mechanical test results of the Ti based alloys.

Mechanical Properties	Ti-10Nb		Ti-20Nb		Ti-30Nb	
Ultimate compressive strength (MPa)	1295	802	618	331	127	48
Yield strength (MPa)	687	136	112	64	46	22
Elastic modulus (GPa)	41	32	28	12	6.7	4.5

## Data Availability

The original contributions given in this work are included in the article, further inquiries can be directed to the corresponding authors.
